# Supplementary material for: Human resources for nephrology in South Africa: A mixed-methods study
Source: PLoS One. 2020 Feb 13;15(2):e0228890. doi: 10.1371/journal.pone.0228890 (PMC7018074; doi:10.1371/journal.pone.0228890)
Supplement: S1 Appendix — (PDF) [file pone.0228890.s001.pdf]

Nephrology Doctor Survey

Dear Colleague

There appears to be insufficient infrastructure and human resources to care for patients with renal disease in South Africa. This survey gathers information on doctors who provide care to patients with end-stage renal disease. By providing accurate data and highlighting deficiencies, we can positively influence the allocation of resources for nephrology and ultimately benefit our patients. Approval for this study has been obtained from Stellenbosch University's Health Research Ethics Committee (ref. S16/05/094).

By completing the survey, you indicate your consent to participate. The survey should take approximately 5 minutes to complete. Any personal details provided will be kept strictly confidential. Thank you for your participation! Please provide us with your email address at the end of the survey if you wish to be sent a summary report.

Yours sincerely
Drs Adriano Pellizzon, Muhammed Hassen (Nephrology Fellows) and Prof Razeen Davids
Stellenbosch University and Tygerberg Hospital

First name

____

Last name

____

Citizenship

- ◯ South African
- ◯ Other (please supply detail)

Citizenship details

____
(Please indicate country of citizenship)

Date of birth

____
(Please enter as D-M-Y. E.g. 12-02-1962)

Sex

- ◯ Male
- ◯ Female

Ethnicity

- ◯ Black (African)
- ◯ Coloured
- ◯ White
- ◯ Indian
- ◯ Other (please provide details)

Ethnicity details

____

Degrees and qualifications

Please indicate the category of your registration with the HPCSA	◯ Adult nephrologist<br>◯ Paediatric nephrologist<br>◯ Specialist physician<br>◯ Specialist paediatrician<br>◯ General practitioner<br>◯ Other (give details)
HPCSA registration category details	__________________________________
What is the scope of your HPCSA registration?	◯ Independent practice<br>◯ Public service<br>◯ Education<br>◯ Retired
Degrees or qualifications obtained - please check all that apply	☐ MBChB or equivalent<br>☐ MMed (Internal Medicine or Paediatrics)<br>☐ FCP (Internal Medicine or Paediatrics)<br>☐ Sub-specialty Certificate in Nephrology (Internal Medicine or Paediatrics)<br>☐ Formal Fellowship in Nephrology (e.g. ISN)<br>☐ PhD<br>☐ MBA<br>☐ Other (please provide details)<br>(Indicate your degrees/qualifications, or closest equivalent. Use "Other" and provide details if necessary.)
Additional details of degrees or qualifications	__________________________________
At which institution did you obtain your basic medical degree?	__________________________________<br>(Indicate the university where you obtained your MBChB or equivalent degree.)
In what year did you obtain your basic medical degree?	__________________________________<br>(Indicate the year when you completed your MBChB or equivalent degree.)
If you have a specialist qualification in internal medicine or paediatrics, at which institution was this obtained?	__________________________________
In what year did you obtain your specialist qualification in internal medicine or paediatrics?	__________________________________<br>(Indicate the year when you obtained your specialist qualification in internal medicine or paediatrics.)
Have you had training in nephrology after obtaining your basic medical degree?	◯ Yes<br>◯ No
At which institution did you obtain your training in nephrology?	__________________________________<br>(Enter N/A if not applicable.)

In what year did you complete the training in nephrology?

____
(Leave blank if not applicable.)

In what year did you first start practicing as a nephrologist?

____

In what year were you first registered as a nephrologist with the HPCSA?

____

If you completed a formal nephrology fellowship (e.g. ISN Fellowship), please indicate the name of the fellowship and the institution at which this was done

____
(E.g. ISN Fellowship at St Michael's Hospital and the University of Toronto)

If you have completed a formal nephrology fellowship, please indicate in what year this was completed

____

If you have completed a formal nephrology fellowship, please indicate the duration of the training in months

____

Location

In which province are you based?

- ◯ Eastern Cape
- ◯ Free State
- ◯ Gauteng
- ◯ KwaZulu-Natal
- ◯ Limpopo
- ◯ Mpumalanga
- ◯ Northern Cape
- ◯ North West
- ◯ Western Cape

In which sector do you mainly practice nephrology?

- ◯ Public sector
- ◯ Private sector
- ◯ Not active (e.g. retired)

Where is your main place of work?

____
(Enter name of hospital, university, dialysis unit, or practice.)

Spectrum of activities

Do you work part-time or full-time?

- ◯ Part Time
- ◯ Full Time

(Part time is normal work hours of less than 40 hours per week)

Please indicate why you are working part time

____

Please indicate the nephrology activities you are currently involved with - select all that apply	☐ Clinical nephrology<br>☐ Teaching<br>☐ Research<br>☐ Administration/management<br>☐ Other (please provide details)<br>☐ Not active (e.g. retired; please provide details)
Additional details of nephrology activities	__________________________________
Please state what percentage of your average working week is devoted to renal clinical work	__________________________________<br>(Please enter a number without the "%" sign. All 5 sections (renal clinical work, non-renal clinical work, research. teaching and administrative work) to total 100%)
Please state what percentage of your average working week is devoted to non-renal clinical work	__________________________________<br>(Please enter a number without the "%" sign. All 5 sections (renal clinical work, non-renal clinical work, research. teaching and administrative work) to total 100%)
Please state what percentage of your average working week is devoted to research	__________________________________<br>(Please enter a number without the "%" sign. All 5 sections (renal clinical work, non-renal clinical work, research. teaching and administrative work) to total 100%)
Please state what percentage of your average working week is devoted to teaching	__________________________________<br>(Please enter a number without the "%" sign. All 5 sections (renal clinical work, non-renal clinical work, research. teaching and administrative work) to total 100%)
Please state what percentage of your average working week is devoted to administrative work	__________________________________<br>(Please enter a number without the "%" sign. All 5 sections (renal clinical work, non-renal clinical work, research. teaching and administrative work) to total 100%)
Total time allocated (%)	__________________________________<br>(The time you have allocated to your different work activities should total 100%.)
If you practice clinical nephrology, please indicate the procedures which you are competent to perform - select all that apply	☐ Insertion of temporary catheters for haemodialysis<br>☐ Insertion of tunneled catheters for haemodialysis<br>☐ Renal biopsies, native kidneys<br>☐ Renal biopsies, transplanted kidneys<br>☐ Bedside insertion of Tenckoff PD catheters<br>☐ Renal ultrasound<br>☐ Ultrasound assessment of the vascular access<br>☐ Other (please provide details)

Additional details of procedures

____

Was your formal training in nephrology adequate to attain proficiency In these fields, or was further independent training required?

	Training adequate	Training inadequate
Insertion of temporary catheters for haemodialysis	○	○
Insertion of tunneled catheters for haemodialysis	○	○
Renal biopsy, native kidney	○	○
Renal biopsy, transplanted kidney	○	○
Bedside insertion of Tenckhoff PD catheters	○	○
Renal ultrasound	○	○
Ultrasound assessment of the vascular access	○	○

If you are based in the PUBLIC sector, do you do any private nephrology work?

○ Yes
○ No

If you are based in the public sector and do some private work, please indicate the nature of this work

☐ Clinical nephrology
☐ Education and training
☐ Research
☐ Administration/management

If you are based in the PRIVATE sector, do you do any public sector nephrology work?

○ Yes
○ No

If you are based in the private sector and do some public sector work, please indicate the nature of this work

☐ Clinical nephrology
☐ Education and training
☐ Research
☐ Administration/management

At what age do you anticipate retirement or stopping work as a nephrologist?

____
(Please indicate age in years)

What factors make you consider retirement at this age?

____

Do you anticipate emigrating from South Africa before retirement?

○ Yes
○ No

Approximately how many years from now do you think you will emigrate

____

What are the reason(s) that are causing you to contemplate emigrating?	☐ Economic opportunities<br>☐ Career development<br>☐ Personal/family reasons<br>☐ Other<br>(Check all that apply.)
Please provide some detail to clarify your choice(s) above.	__________________________________
Which country do you plan to emigrate to?	__________________________________

Work Satisfaction

What are your feelings towards your current work load?	◯ I am happy with my current work load.<br>◯ I am unhappy with a current work load which is excessive.<br>◯ I am unhappy with a current work load which is insufficient.
Are you satisfied with your current remuneration?	◯ Yes<br>◯ No
Are you happy in your current work environment?	◯ Yes<br>◯ No
If not, please briefly state what are the main reasons.	____
Would you recommend nephrology as a career to young physicians/paediatricians or medical students?	◯ Yes<br>◯ No
Do you have any additional comments to add to or clarify any of your above responses?	____
Please indicate whether you would like a summary of the survey findings and whether we may contact you to clarify any of your responses	☐ Yes, please send me a summary of the findings<br>☐ Yes, you may contact me to clarify any of my responses
Email address	__________________________________
Mobile number	__________________________________
Would you be interested in participating in a short focus group discussion at the South African Renal Congress this year regarding nephrology practice in South Africa?	◯ Yes<br>◯ No
