# Supplementary material for: Human resources for nephrology in South Africa: A mixed-methods study
Source: PLoS One. 2020 Feb 13;15(2):e0228890. doi: 10.1371/journal.pone.0228890 (PMC7018074; doi:10.1371/journal.pone.0228890)
Supplement: S2 Appendix — (DOCX) [file pone.0228890.s002.docx]

**S2 Appendix: Prompts used during the focus group discussions**

1. What is the most enjoyable part of your current practice and what is your impression of the nephrology community?
2. What do you think of the numbers of nephrologists in South Africa? Are there too many nephrologists and it is difficult to find work, or are we too few and stretched too thin?
   1. Most nephrologists are in the Western Cape and Gauteng, so the problem may be related to distribution. Can I ask what factors made you decide to practice where you are currently?
3. If you had to think back to your own training, is there anything you would have changed? Was it adequate to prepare you for what you are currently doing?
4. What are major current problems and sources of discontent?
5. Now that we have covered current problems, would any one like to discuss concerns that they have for the future?
6. If we consider retirement at age 65 as standard, has anyone considered retiring earlier or later? And what would those reasons be?
7. A few survey responses indicated that a substantial number of nephrologists were considering emigration. If you have strongly considered emigration, what factors would sway you to stay, and what would convince you go?
8. Anything else which I may have overlooked?
