# Supplementary material for: Human resources for nephrology in South Africa: A mixed-methods study
Source: PLoS One. 2020 Feb 13;15(2):e0228890. doi: 10.1371/journal.pone.0228890 (PMC7018074; doi:10.1371/journal.pone.0228890)
Supplement: S1 Table — Abbreviations: IQR, interquartile range. (DOCX) [file pone.0228890.s003.docx]

**S1 Table.** Demographic details of survey respondents (n=81) working in the public and private healthcare sectors. Abbreviations: IQR, interquartile range.

|  | **Public (n=41)** | **Private (n=40)** | **Total (n=81)** |
| --- | --- | --- | --- |
| **Age (years), median (IQR)** | 47 (39–55) | 46 (41–55) | 46 (40–55) |
| **Sex, n (%)** |  |  |  |
| Male | 24 (59%) | 31 (78%) | 55 (68%) |
| Female | 17 (41%) | 9 (22%) | 26 (32%) |
| **Ethnicity, n (%)** |  |  |  |
| African | 8 (20%) | 3 (7%) | 11 (14%) |
| White | 17 (41%) | 16 (40%) | 33 (41%) |
| Mixed ancestry | 5 (12%) | 2 (5%) | 7 (8%) |
| Indian/Asian | 10 (25%) | 19 (48%) | 29 (36%) |
| Other | 1 (2%) | 0 (0%) | 1 (1%) |
| **Speciality, n (%)** |  |  |  |
| Adult nephrologist | 30 (73%) | 36 (90%) | 66 (81%) |
| Paediatric nephrologist | 11 (27%) | 4 (10%) | 15 (19%) |
| **Province, n (%)** |  |  |  |
| Gauteng | 16 (39%) | 21 (53%) | 37 (47%) |
| Western Cape | 12 (29%) | 10 (25%) | 22 (27%) |
| KwaZulu-Natal | 5 (10%) | 7 (17%) | 12 (15%) |
| Free State | 2 (5%) | 0 (0%) | 2 (2%) |
| Eastern Cape | 4 (10%) | 2 (5%) | 6 (7%) |
| Northern Cape | 1 (3%) | 0 (0%) | 1 (1%) |
| Limpopo | 1 (3%) | 0 (0%) | 1 (1%) |
