# Supplementary figures and images for: Human resources for nephrology in South Africa: A mixed-methods study
Source: PLoS One. 2020 Feb 13;15(2):e0228890. doi: 10.1371/journal.pone.0228890 (PMC7018074; doi:10.1371/journal.pone.0228890)

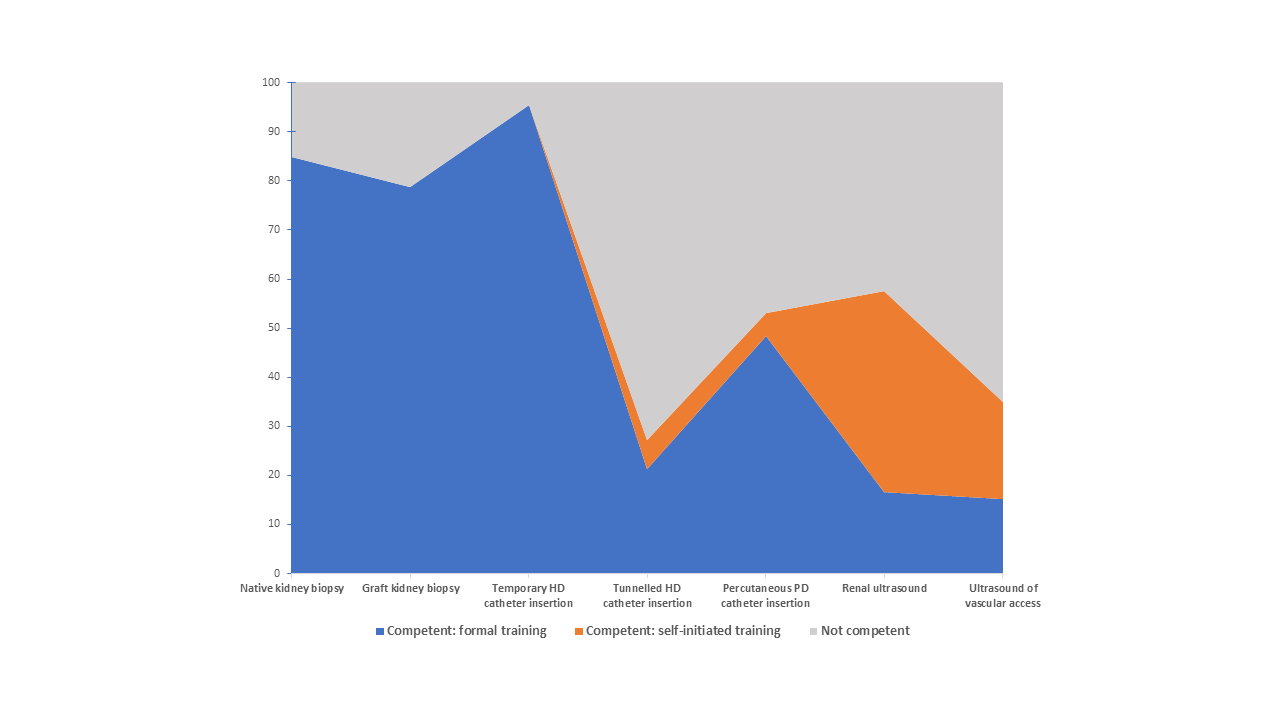

Supplement: S1 Fig — a. Percentage of adult nephrologists (n = 66) reporting competency at various procedures, attained either during formal nephrology training or via self-initiated training. Abbreviations: HD, haemodialysis; PD, peritoneal dialysis. b. Percentage of paediatric nephrologists (n = 15) reporting competency at various procedures, attained either during formal nephrology training or via self-initiated training. Abbreviations: HD, haemodialysis; PD, peritoneal dialysis. (ZIP) [file pone.0228890.s004.zip › S1a_Fig.tif]

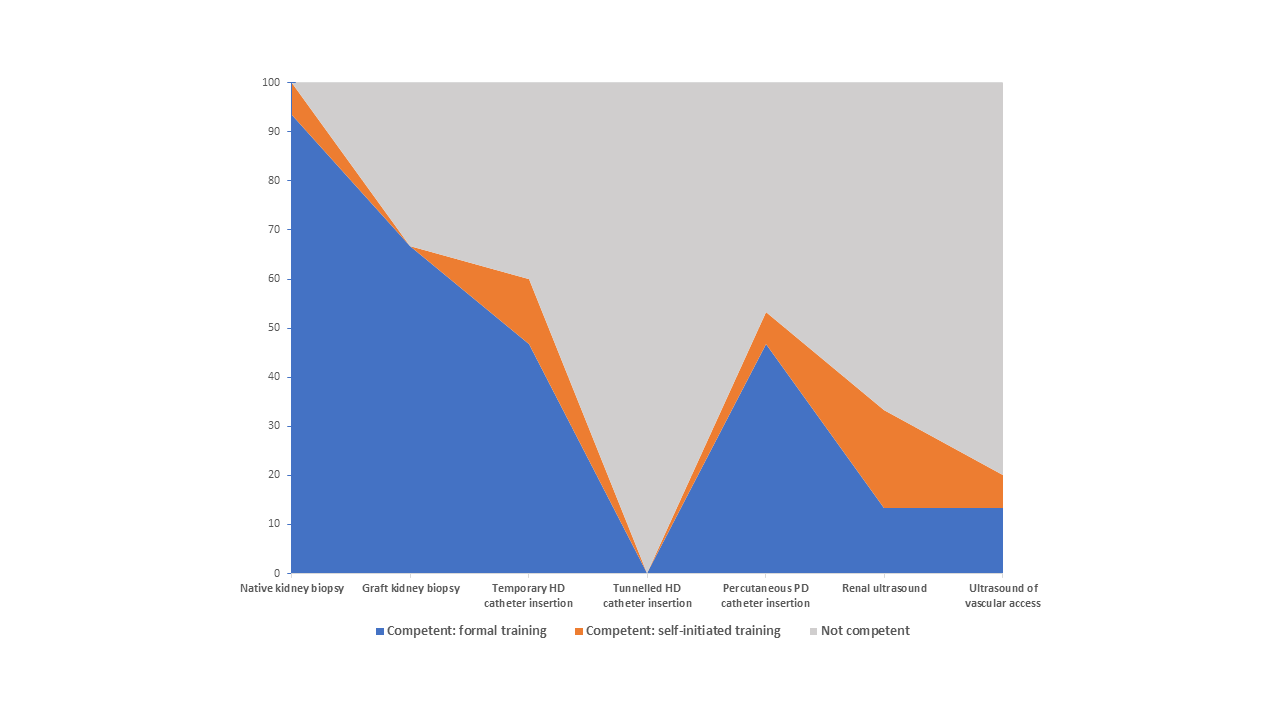

Supplement: S1 Fig — a. Percentage of adult nephrologists (n = 66) reporting competency at various procedures, attained either during formal nephrology training or via self-initiated training. Abbreviations: HD, haemodialysis; PD, peritoneal dialysis. b. Percentage of paediatric nephrologists (n = 15) reporting competency at various procedures, attained either during formal nephrology training or via self-initiated training. Abbreviations: HD, haemodialysis; PD, peritoneal dialysis. (ZIP) [file pone.0228890.s004.zip › S1b_Fig.tif]
